# GIS-based modelling reveals the fate of antlion habitats in the Deliblato Sands

**DOI:** 10.1038/s41598-020-62305-3

**Published:** 2020-03-24

**Authors:** Danijel Ivajnšič, Dušan Devetak

**Affiliations:** 1 Department of Biology, Faculty of Natural Sciences and Mathematics, University of Maribor, Koroška cesta 160, 2000 Maribor, Slovenia; 2 Department of Geography, Faculty of Arts, University of Maribor, Koroška cesta 160, 2000 Maribor, Slovenia

**Keywords:** Ecology, Biodiversity

## Abstract

The Deliblato Sands Special Nature Reserve (DSSNR; Vojvodina, Serbia) is facing a fast successional process. Open sand steppe habitats, considered as regional biodiversity hotspots, have drastically decreased over the last 25 years. This study combines multi-temporal and –spectral remotely sensed data, *in-situ* sampling techniques and geospatial modelling procedures to estimate and predict the potential development of open habitats and their biota from the perspective of antlions (Neuroptera, Myrmeleontidae). It was confirmed that vegetation density increased in all parts of the study area between 1992 and 2017. Climate change, manifested in the mean annual precipitation amount, significantly contributes to the speed of succession that could be completed within a 50-year period. Open grassland habitats could reach an alarming fragmentation rate by 2075 (covering 50 times less area than today), according to selected global climate models and emission scenarios (RCP4.5 and RCP8.5). However, *M. trigrammus* could probably survive in the DSSNR until the first half of the century, but its subsequent fate is very uncertain. The information provided in this study can serve for effective management of sand steppes, and antlions should be considered important indicators for conservation monitoring and planning.

## Introduction

Palaearctic grasslands are among the most threatened biomes on Earth, with one of them – the sand steppe - being the most endangered^1,2^. In Europe, sand steppes and dry grasslands have declined drastically in quality and extent, owing to agricultural intensification, afforestation and abandonment^3–6^. The area of natural grasslands has often been reduced to isolated habitat patches. Moreover, steppes and dry grasslands are considered regional biodiversity hotspots^3,7,8^. Sand steppe in Central and Eastern Europe provides a habitat for many endangered animal species, e.g. the European ground squirrel (*Spermophilus citellus* (Linnaeus, 1766)) and the great bustard (*Otis tarda*, (Linnaeus, 1758)), among the vertebrates^9–11^, and the ground beetle *Carabus hungaricus* Fabricius, 1792, among invertebrates^12–14^. Given their endangerment, sand steppes and dry grasslands rank highly among priority habitats for conservation^15–17^. Unfortunately, conservation efforts are still inadequate, and sand steppes and dry grasslands are further deteriorating. Vulnerable grasslands are shrinking, and their biota declining, with numerous local extinctions having been documented among invertebrates^18–20^.

**Figure 1:**
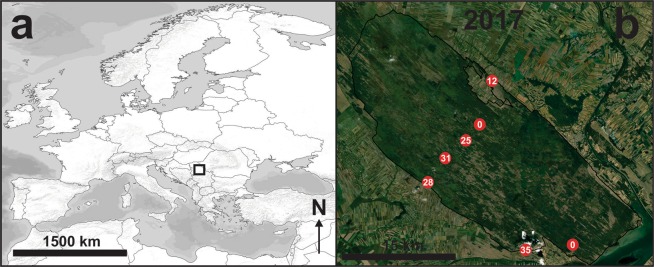
The geographic position of the DSSNR study area (**a**) and the sampling sites with *M. trigrammus* abundance (**b**). Source: Own data and ArcGIS Ocean and Imagery (WGS84) base maps URLs: https://www.arcgis.com/home/item.html?id=52bdc7ab7fb044d98add148764eaa30a, https://www.arcgis.com/home/item.html?id=5ae9e138a17842688b0b79283a4353f6.

The Deliblato Sands Special Nature Reserve (DSSNR) is a protected area in the Vojvodina Province, Serbia (Fig. 1). This geo-morphological formation of eolian origin is located between the river Danube and the southwestern slopes of the Carpathian Mountains. It covers about 300 km^2^ and comprises forest steppe, sand steppe, and small remnants of sand dunes. Maps of the southern part of the Pannonian Plain created in the 18^th^ and 19^th^ centuries^21,22^ reveal that this landscape, which now includes the nature reserve, was at that time dominated by sand, and almost entirely devoid of woody plants. However, a program of intense afforestation of the Deliblato Sands started two hundred years ago, instituted by the authorities of the Habsburg Empire, and caused severe habitat loss. Consequently, the ecological succession (overgrowth) process, here and there interrupted by human activity in the form of vineyard plantation and cattle grazing, before the protection of the DSSNR in 1977^23,24^, has left behind only sporadic or mosaic sandy areas. These areas (habitats comprising bare soil and short-turf grasslands) are inhabited by specific groups of insects, which can be considered as ecological indicators in such environments. From that perspective, the lacewing (Neuroptera) fauna has been surveyed in the area with a special focus on antlion species (family Myrmeleontidae). Antlions are large insects and can therefore be easily identified. Some of them have already been suggested as indicator species for open habitats devoid of trees and bushes in Middle Europe^25^ and South Africa^26,27^, and some antlions are documented as indicators of highly endangered habitats in South Africa^28,29^. In South Africa, a research programme has been undertaken with the goal of monitoring the status of antlions and related lacewings to ensure the conservation and survival of these particularly rich and unique south-African habitats^29^. The results are being used to develop protective measures, such as nature reserve areas with protected species, definition of areas that are inhabited by endangered species, or location and identification of areas that need protection because they are particularly rich in antlions^28,29^. Based on these facts and examples of good practice, we aimed to develop a predictive geospatial model to evaluate the prospects for the DSSNR and its antlions (*Myrmecaelurus trigrammus* (Pallas, 1771) in particular) if conservation actions are not implemented. Geospatial data (environmental and socioeconomic) and complex predictive models designed in the geographic information system environment are gaining acceptance among ecological studies^30^ because they provide applicable results with high value for environmental planners and decision makers in the field of conservation biology^31,32^. It is thus crucial to bridge the gap between geospatial science and ecology to apply innovative spatial approaches to support for effective conservation planning strategies in all vulnerable ecosystems. In that regard, the following research questions were addressed: (1) How rapid is the succession process in the DSSNR? (2) How many open habitats have been lost over the last 25 years, and how many will survive by the end of the century? (3) How will the succession process, triggered by land abandonment and climate change, affect the existing antlion population in future years? And (4), can antlions be considered as an indicator species in the sand steppes for conservation monitoring and planning?

## Material and Methods

### Insect sampling

This study used antlion species, characteristic of sand steppe and grassland habitats. Although most adult antlions are night active (e.g. *Myrmeleon formicarius (*Linnaeus, 1767)*; Euroleon nostras* (Geoffroy in Fourcroy, 1785); *Distoleon tetragrammicus* (Fabricius, 1798)), they can also be easily collected during the day with an insect net^33^. Collecting larval antlions in the DSSNR study area would not be successful, since only the larvae of *Myrmeleon inconspicuous* (Rambur, 1842)*, M. formicarius* and *E. nostras* construct pits, while the rest of the species listed in this study are non-pit-builders. We sought to use a robust sampling technique that could be carried out by employees of similar protected areas around the World in order to ensure the replicability, transferability, and above all, applicability of our results. Thus, random sweep-netting turned out to be the most convenient sampling method. Moreover, the focus of this research is not oriented towards species composition change on selected sites but to the currently most abundant diurnal antlion species *Myrmecaelurus trigrammus* (Pallas, 1771) (hereafter *M. trigrammus*), a potential indicator for regionally rare open sandy grassland habitats. Such an approach minimizes the sampling error and thus excuses the robust methodological procedure employed.

Adult antlions were collected in the study area within the DSSNR in July 2016. Open grassland habitats (N = 7) of varied size were selected and GPS recorded with a GARMIN GPS device (Fig. 1b). The abundance of antlions was determined by counting captured individuals on these sites, collected by the sweep-netting method for one hour by three persons in a plot of 50 m × 100 m. All open grassland habitats were visited at the same hour (12:00) of the day. After identification to a species level, specimens were returned to their natural habitat. For antlion identification, we used the keys in Badano and Pantaleoni^34^ and Aspöck *et al*.^35^ and some collected larvae excavated by spoon. However, the most abundant antlion species (occurring in all sampled open grassland habitats) – *M. trigrammus -* was then chosen for assessment of its current and potential future abundance in the whole DSSNR study area.

### Time series file development

To detect the successional dynamics across the whole study area of the DSSNR, freely available cloud-free LANDSAT multispectral imagery was obtained from the EarthExplorer web platform (https://earthexplorer.usgs.gov/). Data from 1992 to 2017 were downloaded and converted to TerrSet’s^36^ time series (TSF) format. However, for some time windows (1993, 1995–1999, 2002, 2004, 2008 and 2012), satellite images were not available, owing to cloud cover or sensor error in the target months (July and August). Thus, LANDSAT 4, 5, 7 and 8 products were applied to develop the first time series database (NDVI) containing 17 time frames.

Environmental and/or anthropogenic factors can accelerate or decelerate a succession process. Modern climate change, triggered by human activity, can be considered as a key determinant from both perspectives. We tested whether climate variables contribute significantly to the succession trend in the DSSNR study area. The CHELSA geospatial climate database (http://chelsa-climate.org/) was used to produce annual mean air temperature (T) and precipitation variables (RR). These were gathered for almost the same period as the satellite data (1993–2016, [2017 not yet available on the CHELSA database]) in order to compare climate variability and biomass change dynamics in the DSSNR. For that purpose, a second time series database (current climate) was developed.

In the next step, future climate variables (Coupled Model Intercomparison Project [CMIP5]) were prepared for the study area by considering five (5) low interdependent global climate models (CCSM4, CESM1-BGC, GFDL-ESM2G, MIROC5 and MPI-ESM-MR), two representative concentration pathway climate change scenarios (RCP4.5 [580–720 ppm CO_2_eq in the atmosphere and a 1.7–3.2 °C air temperature increase by 2100, relative to the 1850–1900 period] and RCP8.5 [>1000 ppm CO_2_eq, 3.2–5.4 °C]) and two future time windows (2050 [2041–2060] and 2075 [2061–2080]). These models were selected to meet the requirements for capturing a decent amount of uncertainty in climate model projections^37^. Additionally, multi-model ensemble T and RR variables for the DSSNR study area were calculated for each future time window and emission scenario (RCP). Thus, a third time series database (future climate) was developed. All three geospatial time series datasets were combined in a geospatial model to estimate and predict the potential development of open grassland habitats and the abundance of one linked antlion species *M. trigrammus* in the DSSNR.

### A pixel level regression

Initially, the visible red and near-infrared bands (30 m horizontal resolution) were used to calculate the Normalized Difference Vegetation Index (NDVI) for each time window (17) by applying the VEGINDEX tool in Terrset software^36^. NDVI is a vegetation index commonly used in ecological studies because it carries information about vegetation density or biomass and minimizes the topographic effect, while producing a linear measurement scale ^38,39^. The NDVI value for a given pixel always returns a number ranging from minus one (−1) to plus one (+1); however, the absence of green leaves gives a value close to zero, whereas zero means no vegetation, and values close to +1 (0.8–0.9) indicate the highest possible density of green leaves^38^.

**Figure 2:**
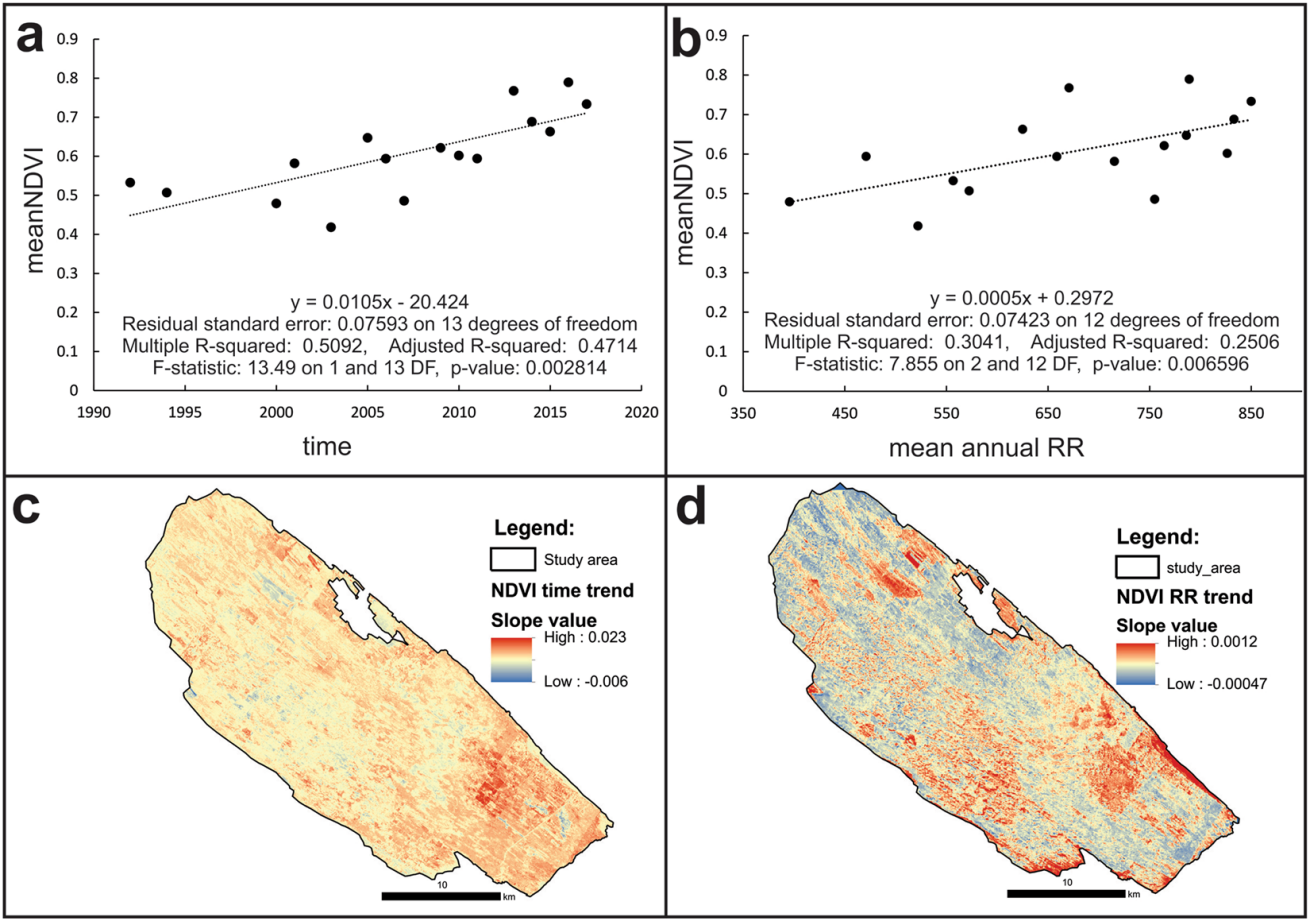
The NDVI-time (**a**) and NDVI-RR (**b**) relationship (1992–2017) and the corresponding spatial slope coefficients (**c**,**d**) in the DSSNR study area.

Prior to the pixel level regression analysis, mean NDVI values, mean T and mean RR values for 16 time windows (from 1993 to 2016) were calculated for the whole DSSNR study area by applying the zonal statistics tool in ArcGIS^40^. Thus, the NDVI-time, NDVI-RR and NDVI-T relationships were obtained. Since time and RR had a significant linear impact on the NDVI dynamics in the DSSNR study area (Fig. 2a,b), both were used as NDVI predictors on a pixel level (30 m^2^) basis. Before that, all the missing geospatial data were statistically interpolated by using the linear temporal interpolation algorithm within the Earth Trends Modeler (ETM) module in TerrSet^36^. In order to identify the speed of the overgrowing process, the linear NDVI trends with corresponding slope (β coefficient), R^2^ and p-values were calculated with the predictors time (Fig. 2c) and RR (Fig. 2d) for each pixel (30 m^2^) in the study area by applying the Series Trend Analysis tool within ETM^36^. Both NDVI slope variables (time- and RR-derived) were then combined with the weighted sum algorithm by considering corresponding weights according to the residual standard error values resulting from the current NDVI-time and NDVI-RR relationship (Fig. 2a,b). Thus, the final combined NDVI slope coefficient enabled not only the objective estimation of the possible disappearance of open habitats in the study area under several climate change scenarios, but also the estimation of potential future abundance of the target species *M. trigrammus*, which is linked to those open areas within the DSSNR. Finally, future time-derived and RR-derived NDVI variables were calculated and then summed with corresponding weights for scenarios RCP4.5 and RCP8.5 for the years 2050 and 2075.

### Geospatial modelling

A geospatial model was developed to estimate future abundance of *M. trigrammus* in the DSSNR study area under climate change scenarios RCP4.5 and RCP8.5 for the years 2050 and 2075. Two ecogeographical (EG) predictor variables (mean NDVI in each open habitat [mNDVI] and mean distance to forest in each open habitat [mD2F]) were calculated to properly fit a Poisson regression model. To obtain these two EG predictors, the following methodological procedures were completed: (i) determination of open and closed habitats, (ii) vectorization of open and closed habitats, (iii) distance matrix calculation and, finally, (iv) zonal statistics calculation.

Open and closed habitats were determined based on 2017 NDVI values. Thirty (30) spatial signature polygons were developed across the study area for each type. The signatures were used for supervised image classification by applying the maximum likelihood algorithm in TerrSet. The threshold NDVI values for open and closed habitats were determined with these spatial signature polygons. Next, the raster layers representing open and closed habitats were converted to polygons with the purpose of calculating mNDVI and mD2F variables per open habitat polygon in the final step. To do so, a variable for distance to closed habitat (forest) was developed with the Euclidean distance tool in ArcGIS^40^. The zonal statistics tool was used in the final step to calculate the mean values per open habitat polygon in the study area.

**Figure 3:**
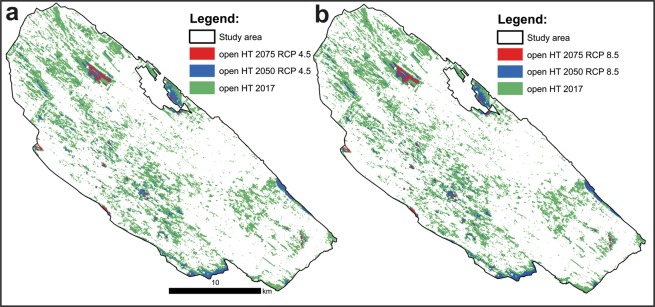
The potential development of open grassland habitats in the DSSNR study area under RCP4.5 (**a**) and RCP8.5 (**b**) scenarios.

Both EG variables were tested for possible significant correlation across the study area with the Band Collection statistics tool in ArcGIS to avoid possible variable redundancy^41^. Finally, a Poisson regression model with those two EG predictors was developed to estimate the abundance of *M. trigrammus* in the DSSNR study area and to predict its future abundance by considering the speed of the succession process triggered by land abandonment and climate change, manifested in the mean annual precipitation variability. The Poisson model was fitted in R statistical software^42^ by applying the glm function. In addition, the residual deviance parameter was used to perform a goodness of fit test for the overall model. The residual deviance is the difference between the deviance of the current model and the maximum deviance of the ideal model, where the predicted values are identical to those observed. Therefore, if the residual difference is small enough, the goodness of fit test will not be significant, indicating that the model fits the data. After assuring a properly specified regression model, future EG predictors (2050 and 2075) were calculated by implementing the slope coefficients of the combined time- and RR-derived NDVI trends for each RCP scenario. Consequently, the geographic position and total area of open habitat across the study area in selected future time windows and climate change scenarios were estimated (Fig. 3). The Poisson regression equation then enabled the prediction of potential abundance of *M. trigrammus* in open areas possibly still existing in the DSSNR by 2050 and 2075.

Owing to the small size of patches representing open grassland areas in future time windows, we designed a hexagon (width = 1000 m) network over the DSSNR study area with the Repeating shapes tool^43^ within ArcGIS^40^. The predicted abundance of *M. trigrammus* was then summed for each hexagon in order to improve the visualization and interpretation of the results.

## Results

### Antlion assemblages in the Deliblato sands

Ten (10) antlion species have been recorded in the DSSNR; among them, three species (*Palpares libelluloides* (Linnaeus, 1764), *Acanthaclisis occitanica* (Villers, 1789) and *Nohoveus punctulatus* (Steven in Fischer v. Waldheim, 1822)) are suspected of being locally extinct in Serbia (Table 1). Among recently detected antlions, three species frequently occurred during the survey in July 2016: *Myrmecaelurus trigrammus*, *Creoleon plumbeus* (Olivier, 1811) and *Myrmeleon inconspicuus*. One of these, *M. trigrammus*, was considered as the most appropriate for geospatial modelling, given that it had the highest abundance in each sampled open grassland habitat. However, all three frequently occurring antlions are indicators of sand steppes in the Pannonian Basin. Based on these results and existing literature, we estimated the conservation status of antlion species in Serbia (Table 1).

**Table 1:** Antlion (Neuroptera, Myrmeleontidae) species recorded in the Deliblato sands, Serbia.

Species	Record from DSSRN; frequency of occurrence	Ecological demands	Indicator species for	Estimated conservation status in Serbia
*Palpares libelluloides* (Linnaeus, 1764)	1982 – 1 individual collected in DSSRN	Mosaic landscape: grassland/shrub	—	Ex?
*Acanthaclisis occitanica* (Villers, 1789)	Only records before WWI existed in DSSRN	Sand steppe, sand dunes	Sand steppe, sand dunes	Ex?
*Myrmecaelurus trigrammus* (Pallas, 1771)	Common species in DSSRN, but rare in SRB	Sand steppe, sand dunes	Sand steppe, sand dunes	V
*Nohoveus punctulatus* (Steven in Fischer v. Waldheim, 1822)	Only records before WWI existed in DSSRN	Sand steppe, sand dunes	Sand steppe, sand dunes	Ex?
*Myrmeleon formicarius* Linnaeus, 1767	Rare in DSSRN, but common in SRB	Euryoecious species	—	Not endangered
*Myrmeleon inconspicuus* Rambur, 1842	Common species in DSSRN, no records in other parts of SRB	Sand steppe, sand dunes	Sand steppe, sand dunes	V
*Euroleon nostras* (Geoffroy in Fourcroy, 1785)	Rare in DSSRN, but common in SRB	Euryoecious species	-	Not endangered
*Distoleon tetragrammicus* (Fabricius, 1798)	Rare in DSSRN	Thermophilous euryoecious species	—	Not endangered
*Creoleon plumbeus* (Olivier, 1811)	Common species in DSSRN, no records in other parts of SRB	Sand steppe, sand dunes	Sand steppe, sand dunes	V
*Megistopus flavicornis* (Rossi, 1790)	1988 – 2 individuals collected in DSSRN	Relative euryoecious species	—	DD

Legend: DSSNR – Deliblato Sands Special Nature Resrve; SRB – Serbia; Ex? – presumably extinct; DD – deficient data; V – vulnerable; WWI – World War One.

### Pace of succession

Historical maps (The First and the Second military survey of the Habsburg Empire) revealed that our study area was practically a sandy desert-like environment by the end of the 18^th^ century and was sparsely covered with wooded plants by the second half of the 19^th^ century.

However, over the last 25 years, the forest has constantly progressed and is slowly fragmenting the existing open grassland habitats in the DSSNR (Fig. 1b). The pixel level regression analysis revealed the succession dynamics in detail. Time and mean annual precipitation (RR) are key natural determinants controlling the speed of the succession process in the study area (Fig. 2). Thus, the NDVI-time and NDVI-RR relationship, and the developed geospatial time series datasets (NDVI, current climate and future climate), enabled the objective estimation of future open grassland habitat distribution in the DSSNR.

In 2017, open grassland habitats covered 13815 hectares (23.5%). By considering the RCP4.5 climate scenario, open grassland habitats in the DSSNR study area could occupy nine times less area (1580 Ha or 2.69%) by 2050 or only 274 hectares (0.47%) by 2075 (Fig. 3a). The RCP8.5 scenario, the most pessimistic from the greenhouse gas emission perspective, offered a slightly better outcome for open areas in the DSSNR. Nevertheless, by 2050 these could shrink by 11.7% compared to the situation in 2017, covering 1614 hectares by then. The fragmentation process of open grassland habitats in the study area could potentially reach an alarming state by 2075, if RCP8.5 or a similar emission scenario is realized, covering an area of only 284 hectares (0.48%) (Fig. 3b).

### Ecogeographical (EG) variables and the Poisson regression model

Since vegetation density and open habitat size are key predictors of *M. trigrammus* abundance in the DSSNR study area, these were developed for the present and for each future time window and RCP (greenhouse gas emission) scenario. The mNDVI value per open habitat polygon is not expected to change much over time. If mNDVI values exceed the open habitat threshold value, they are automatically considered as indicating a closed habitat. However, some variability in this EG variable still exists, where open habitats with low mNDVI values (low biomass or density) are becoming slowly overgrown with time but are still within the open habitat threshold value. In contrast, the change in the mD2F EG variable is much more intensive. Because open habitats are losing area and are increasingly invaded by forest, the distance variable is taking over lower values in each successive future time window.

However, the weak correlation (r < 0.3) between the two predictor variables allowed the proper fitting of a Poisson regression model (Table 2), which was later applied in the geographical space to predict the potential future abundance of *M. trigrammus* across the study area. The insignificant goodness-of-fit chi-squared test (p > α, α = 0.05), based on residual deviance, additionally proved that the model was properly specified. Both predictor variables had a significant influence on the dependent variable (*M. trigrammus* abundance). As expected, estimates (log value) were negative, since higher abundance was clearly linked to larger open areas that were less densely vegetated.

**Table 2:** Poisson regression summary.

Coefficients	Estimate	Std. Error	z value	Pr(>|z|)	
(Intercept)	16.37584	4.13682	3.959	7.54E-05	***
mNDVI	−21.89299	6.92786	−3.16	0.00158	**
mD2F	−0.01534	0.00499	−3.074	0.00212	**
Null deviance: 101.4933 on 6 degrees of freedom					
Residual deviance: 7.6274 on 4 degrees of freedom					
AIC: 38.879					

### The future of the antlion Myrmecaelurus trigrammus in the DSSNR

**Figure 4:**
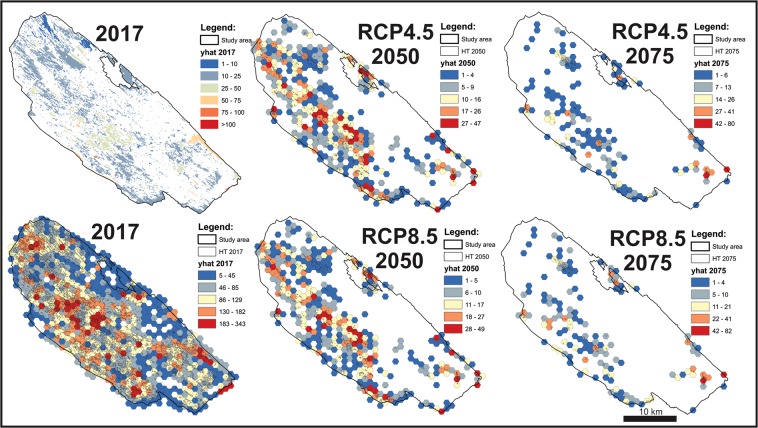
The potential future abundance of *M. trigrammus* in the DSSNR study area under RCP4.5 and RCP8.5 climate change scenarios.

According to the model, most (67%) of the currently existing open habitats in the study area can potentially host *M. trigrammus*, with an abundance of 10–25 individuals/5000 m^2^/hour. 17% of open habitats offer suitable conditions for 25–50, and only 4% of the study area is identified as having the optimal suitability for *M. trigrammus*. On the remaining 11% of open habitat, approximately 1–10 individuals/5000 m^2^/hour can be expected. These abundance categories will change drastically with the intensive successional process, driven by land abandonment and climate change (Fig. 4). By summing *M. trigrammus* abundance per created hexagon network, the spatial pattern and its future development are more recognizable. Both considered RCP scenarios assume a drastic decrease of most suitable habitats by 2050, which could then potentially host up to 7 times fewer *M. trigrammus* individuals per hexagon. However, the spatial abundance pattern of *M. trigrammus* could remain rather stable by 2050, concentrated in the central part of the study area, but could change completely by 2075. More *M. trigrammus* abundance hot spots are then expected in the southern part of the DSSNR. Nevertheless, the target species could face an extremely high extinction risk in the second half of the 21^st^ century, since both RCPs indicate that most of the possibly still existing, open grassland habitats could have an *M. trigrammus* abundance of only 1 to 13 individuals per hexagon.

## Discussion

Massive afforestation of the DSSNR during the last two centuries had strong deleterious effects on the ecosystem composition, where more than three-quarters of the former natural habitat has been lost. While significant erosion control is a feasible positive effect, habitat loss is likely to be a negative consequence of afforestation in grasslands and sand dune ecosystems^44^. Habitat loss, as a consequence of afforestation and natural succession, is a crucial threat to the DSSNR. Maintenance of a steady state within an ecosystem requires continuous monitoring of biodiversity and ecosystem function^45^. In steppe grasslands, this can be realized by monitoring indicator species of antlions, whose population levels are easily measured. Antlions are sand- and grassland dwellers with behavioural, morphological and physiological adaptations for their environment^28^. They could have a substantial value for conservation needs^25,28,29^. It is widely accepted that a nationally rare or threatened species present in a surveyed area has a higher conservation value than a ubiquitous species because it contributes more to regional biodiversity than a common species^46,47^. Dry grasslands and sand steppes harbour a high proportion of these insects, whose larvae require a sandy microhabitat for completion of their larval development.

Based on our results, *M. trigrammus* should be regarded as an indicator species, characteristic of the sand steppe in the southern rim of the Pannonian Plain. Outside the Mediterranean, another two antlion species (*C. plumbeus* and *M. inconspicuus*) commonly occur in the sand steppe of the Pannonian Plain, in sand dunes or the remnants of formerly widespread sand dunes. Both have considerable potential to be considered as other ecological indicator species in these environments. However, they are rare in adjacent countries^25,48–56^. Two antlion species, *A. occitanica* and *N. punctulatus*, which have not been found in the DSSNR since the beginning of the 20^th^ century, are declining throughout their range and are near extinction in some European countries^54,57,58^. One rare species, *P. libelluloides*, was found in the DSSNR only once; it is an insufficiently known antlion, probably extinct in the reserve^59^.

Our model predicts that natural succession in the form of afforestation could be completed within a 50-year period. This prediction leads to the conclusion that all grassland-linked taxa, including the antlions typical of the sand steppe, could become locally extinct. However, this process could be decelerated by permanent temperature increase and precipitation reduction, trends detected in the last half of the century^60^. We tested this assumption by integrating past, recent and future geospatial climatic data with the remotely sensed biomass change dynamics in the DSSNR study area. Some direct climate change impact on the target species *M. trigrammus* and others, can be expected in the coming years, but these will be negligible compared to those climate drivers influencing the succession process. The key factor in this case is mean annual precipitation, which is positively correlated with the biomass gain rate in the study area. Although the selected global climate models and emission scenarios predict less precipitation for the DSSNR in the second half of the 21^st^ century, open grassland habitats could reach an alarming fragmentation rate by 2075. Nature conservation actions are urgently needed if we want to preserve the DSSNR’s current biodiversity.

From this perspective, a partial restoration of the DSSNR would be the only way to prevent the disappearance of natural habitats along with their fauna and flora. These restored open habitats must then, of course, be properly maintained. In this regard, fires have a positive effect in maintaining grassland habitats. Natural fire is considered as a natural disturbance that has many benefits for grassland ecosystems^61^. It can be a significant evolutionary force, which has driven the evolution of species^62^. Many plant species require natural fire to germinate and reproduce. Thus, a carefully chosen and controlled fire regime has a role in maintaining biodiversity in grassland or savanna ecosystems^63^. Moreover, controlled or prescribed burning is a tool that is receiving additional attention in ecosystem studies and protected area management. Dormant-season prescribed burning was applied to grasslands in East-Hungary in order to test its effects from the nature conservation perspective^64^. This experiment resulted in changed soil characteristics along with changes in plant biomass and composition. On the burnt sites, soil organic matter, pH, potassium and phosphorous did not change significantly, but soluble salt content increased drastically^64^. Other positive effects were also detected: an increased number of flowering shoots and plant diversity, along with stable arthropod abundance and diversity^64^.

In contrast to grazing, which is accompanied by nitrification of the soil, consequently influencing grassland species composition^65^, forest fires have no effect on that process. Indeed, forest fires and cattle grazing did occur in the past in the DSSNR^23,24^. Finally, we can conclude that controlled burning could be the most appropriate grassland management tool in the case of the DSSNR, because it supports plant diversity and does not threaten the invertebrate fauna; such conservation actions should be implemented there as soon as possible, in order to avert high local extinction risk and the worst-case scenario.

## Data Availability

All the data and results in the form of tables and maps are available on request.
